# Co-infection with *Legionella* and SARS-CoV-2, France, March 2020

**DOI:** 10.3201/eid2711.202150

**Published:** 2021-11

**Authors:** Camille Allam, Alexandre Gaymard, Ghislaine Descours, Christophe Ginevra, Laurence Josset, Maud Bouscambert, Laetitia Beraud, Marine Ibranosyan, Camille Golfier, Arnaud Friggeri, Bruno Lina, Christine Campèse, Florence Ader, Sophie Jarraud, COVID-19 diagnosis HCL consortium

**Affiliations:** Hospices Civils de Lyon, Lyon, France (C. Allam, A. Gaymard, G. Descours, C. Ginevra, L. Josset, L. Beraud, M. Ibranosyan, C. Golfier, A. Friggeri, B. Lina, F. Ader, S. Jarraud);; Université de Lyon, Lyon (C. Allam, A. Gaymard, G. Descours, C. Ginevra, L. Josset, M. Ibranosyan, B. Lina, F. Ader, S. Jarraud);; Centre International de Recherche en Infectiologie, Lyon (C. Allam, A. Gaymard, G. Descours, C. Ginevra, L. Josset, B. Lina, F. Ader, S. Jarraud);; Santé Publique France, Saint-Maurice, France (C. Campèse)

**Keywords:** coronavirus disease, COVID-19, Legionnaires’ disease, Legionella, pneumonia, respiratory infections, severe acute respiratory syndrome coronavirus 2, SARS-CoV-2, viruses, zoonoses

## Abstract

We describe a March 2020 co-occurrence of Legionnaires’ disease (LD) and coronavirus disease in France. Severe acute respiratory syndrome coronavirus 2 co-infections were identified in 7 of 49 patients from LD case notifications. Most were elderly men with underlying conditions who had contracted severe pneumonia, illustrating the relevance of co-infection screening.

The coronavirus disease (COVID-19) pandemic spread to France in mid-February 2020 (*1*). Co-infections have been described in patients with COVID-19 (*2*,*3*), but only 3 co-infections with *Legionella* have been reported (*4*–*6*). We report 7 cases of severe acute respiratory syndrome coronavirus 2 (SARS-CoV-2) and Legionnaires’ disease (LD) co-infections in France during March 2020.

## The Study

In France, LD surveillance is based on mandatory notifications to Santé Publique France, the national public health agency. To evaluate LD and COVID-19 co-occurrence, we retrospectively studied all LD case notifications with symptom onset during March 2020 and included cases in which patients had clinical or radiologic signs of pneumonia combined with *Legionella* culture, positive *Legionella* PCR from broncho-pulmonary secretions, or positive *Legionella pneumophila* serogroup 1 urinary antigen test (UAT) results. There were 65 LD case notifications in March 2020 compared with 79 in March 2019. To evaluate the number of UATs, which are performed in 96% of LD cases (*7*), we contacted the 59 reporting laboratories (in 47 cities), 33 of which sent the relevant data. The number of UATs increased 2.5-fold (interquartile range: 1.6–2.8) from 3,203 in March 2019 to 8,004 in March 2020. Data obtained from 6 major UAT suppliers indicated a similar 2.1-fold (interquartile range 1.52–14.8-fold) increase in tests sold to laboratories in France, from 33,378 in March 2019 to 65,072 in March 2020. Despite these increases, the number of LD case notifications was 18% lower in March 2020 than in March 2019.

Among the 65 patients from the case notifications, 49 were tested for both LD and COVID-19 and 12 for LD only; no information was available for 4. The frequency of proven LD/COVID-19 co-infection was 14.3% (7/49). This finding may be an overestimate because COVID-19 incidence was <5 cases/100,000 persons in the region of residence of the 16 patients not tested at the time of symptom onset; actual co-infection frequency could be from 10.8% (7/65) to 14.3% (7/49). 

Most patients (4/7) with co-infection lived in the Grand Est region, the area in France with the most COVID-19 cases during the study period and a region that usually reports a high number of LD cases. Median patient age was 72 years (range 37–83 years); male-to-female ratio was 6:1 (Table), higher than for the overall COVID-19–infected population (*8*). Of interest, the male-to-female ratio for LD has elsewhere been reported as ≈3:1 (*7*), similar to the ratio observed in the LD-positive/COVID-19–negative cases.

**Table:** Patient demographics, underlying conditions, and risk exposures for patients with LD, with and without co-occurring COVID-19, France, 2020*

Patient no.	LD-positive, COVID-19–negative	LD- and COVID-19–positive	LD- and COVID-19–positive patient details						
			1	2	3	4	5	6	7
Demographics†									
Sex	M:F ratio 23:18	M:F ratio 6:1	M	M	M	F	M	M	M
Age, y	Median (range), 67 (36–96)	Median (range), 72 (37–83)	72	71	71	83	73	73	37
ICU admission	10/31 (32)	7/7 (100)	Y	Y	Y	Y	Y	Y	Y
Outcome†									
Recovered	7/42 (17)	1/7 (14)	N	N	N	Y	N	N	N
Death	3/42 (7)	2/7 (29)	N	N	Y	N	N	Y	N
Ongoing disease	32/42 (76)	4/7 (71)	Y	Y	N	N	Y	N	Y
Underlying conditions									
Corticotherapy‡	1/42 (2)	2/7 (29)	Y	Y	N	N	N	N	N
Other immunosuppression‡	5/42 (12)	0/7 (0)	N	N	N	N	N	N	N
Smoking‡	15/42 (36)	2/7 (29)	N	Y	Y	N	N	N	N
Cardiovascular diseases	1/42 (2)	6/7 (90)	Y	Y	Y	Y	Y	Y	N
Chronic respiratory disease	4/42 (10)	1/7 (14)	N	Y	N	N	N	N	N
Chronic renal insufficiency	1/42 (2)	1/7 (14)	N	N	N	N	N	Y	N
Diabetes‡	10/42 (24)	2/7 (29)	N	Y	N	N	Y	N	N
Hemopathy or cancer‡	3/42 (7)	2/7 (29)	Y	N	Y	N	N	N	N
≥1 underlying condition	25/42 (60)	6/7 (86)	Y	Y	Y	Y	Y	Y	N
Exposures§									
Hospital‡	1/42 (2)	2/7 (0)	Y	Y	N	N	N	N	N
Nursing home‡	2/42 (5)	0/7 (0)	N	N	N	N	N	N	N
Travel associated/tourism‡¶	11/42 (26)	1/7 (29)	N	Y	N	N	N	N	N
Professional exposure‡#	2/42 (5)	0/7 (0)	N	N	N	N	N	N	N
Other exposure‡**	0/42 (0)	1/7 (29)	N	N	N	N	Y	N	N
≥1 exposure	14/42 (33)	3/7 (43)	Y	Y	N	N	Y	N	N

*Values are no. patients/total no. in category (%) except as indicated. COVID-19, coronavirus disease; ICU, intensive care unit; LD, Legionnaires’ disease. †Demographics and outcome at the LD notification date. ‡Elements indicated in mandatory LD notifications. LD risk exposures had to be reported if they occurred ≤14 d before LD symptom onset. §Exposures were indicated in LD mandatory notifications. ¶Travel-associated/tourism exposures include hotels, holiday resorts, rental houses, and cruises. #Professional exposures include using public showers during work hours. **Other exposure: in-house plumbing.

At hospital admission, co-infected patients had more underlying conditions; 6 (86%) of 7 patients had ≥1, compared with 25 (60%) of 42 LD-positive/COVID-19–negative patients (Table). For cardiovascular diseases, the proportions were 6 of 7 among co-infected and 1 of 42 among LD-positive/COVID-19–negative patients. Sources of LD exposure were reported in case notifications for 3 (43%) of 7 co-infected versus 14 (33%) of 42 non–co-infected patients; the proportions of exposure sources reported was similar between the 2 groups. Despite the implementation on March 15 of the COVID-19 national lockdown in France, halting travel, 12 (24%) of 49 exposures from the LD notifications were travel associated, a ratio similar to that in a 2017 report (*9*). Therefore, the decrease in LD cases observed in March 2020 cannot be explained by decreased travel.

**Figure 1:**
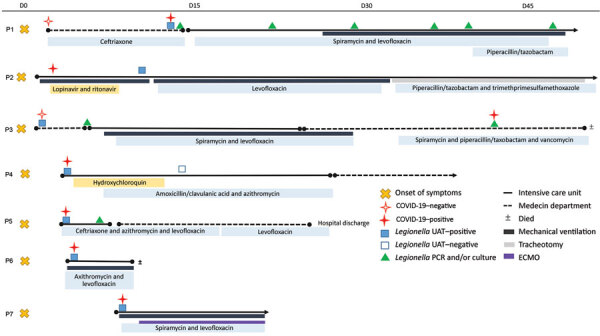
Timeline of first Legionnaires’ disease symptoms among 7 Legionnaires’ disease/COVID-19 co-infected patients, France, March 2020. COVID-19, coronavirus disease; D, day; ECMO, extracorporeal membrane oxygenation; UAT, urinary antigen test.

Community-acquired LD and COVID-19 were diagnosed at hospital admission in 5 of 7 patients with both infections. For patient 2 (Table), whose symptoms started 48 hours before admission, UAT was not performed until 7 days after admission. Hospital-acquired COVID-19 was suspected in patient 3 because he initially tested negative but was positive after a 4-week hospitalization (Figure 1). All 7 co-infected patients required admission to an intensive care unit (ICU; median stay 13 days,; range 2–34 days). In contrast, only 10 (32%) of 31 of LD-positive/COVID-19–negative patients required ICU, similar to LD-only patients in previous reports (*7*).

At admission, all 7 co-infected patients had hyperthermia, 6 had cough or dyspnea, and 2 had neurologic symptoms. Five patients needed orotracheal intubation for a median of 13 days (range 3–30 days); acute respiratory distress syndrome developed in 4 patients, and 1 required extracorporeal membrane oxygenation (Figure 1). The median follow-up was 24 days (range 2–34 days); 2 (29%) of 7 patients died, similar to death rates for known ICU LD patients (*7*,*10*) and severe COVID-19 patients (*8*). Three (7%) of 42 LD-positive/COVID-19–negative patients died, consistent with overall LD death rates (*7*). Patient 6 died within 3 days after co-infection diagnosis. Patient 3 had progressive pulmonary deterioration and died 6 days after COVID-19 diagnosis. First-line LD treatment was appropriate for all patients; 2 patients received COVID-19 treatment (Figure 1).

**Figure 2:**
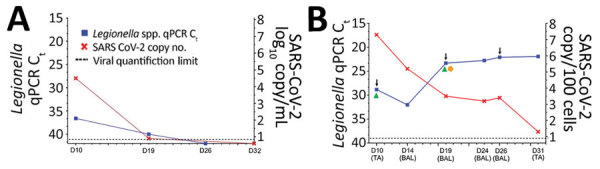
Timeline of detection of SARS-CoV-2 and *Legionella* in patient 1, a 71-year-old man receiving chemotherapy for multiple myeloma, France, March 2020. A) Serum *Legionella* DNA load estimated by qPCR C_t_ and SARS-CoV-2 RNA load expressed as the number of log_10_ RNA copies/mL serum. B) Pulmonary *Legionella* DNA load estimated by qPCR C_t_, targeting the 16sRNA gene (R-DiaLeg, Diagenode, https://www.diagenode.com) and SARS-CoV-2 RNA load (Institut Pasteur, Paris protocol). Arrows indicate dates of computed tomography scans, green triangles indicates dates of positive *Legionella* cultures, and orange circle indicates date of lung microbiome testing. Values are normalized according to cellular quantification using the CELL Control r-gene kit (bioMérieux, https://www.biomerieux.com) and expressed as the number of log_10_ RNA copies/100 cells from pulmonary TA and BAL. BAL, broncho-alveolar lavage; C_t_, cycle threshold; D, days after onset of symptoms; qPCR, quantitative PCR; SARS-CoV-2, severe acute respiratory syndrome coronavirus 2; TA, tracheal aspirations.

The longitudinal follow-up of patient 1, a 71-year-old man receiving chemotherapy for multiple myeloma, may help decipher the kinetics of each pathogen load. Hospitalized for fever (39°C) and productive cough, he required ICU admission on day 9 because of acute respiratory distress syndrome. A thoracic computed tomography scan found left lobar atelectasis, multiple ground-glass opacities compatible with COVID-19, and pleural effusion suggesting possible bacterial infection. Results of UAT and nasopharyngeal SARS-CoV-2 reverse transcription PCR were both positive. On day 10, a serum sample was PCR positive for both SARS-CoV-2 and *Legionella* (Figure 2, panel A); each pathogen has individually been associated with COVID-19 (*11*) and LD (*12*) severity. Beginning on day 10, longitudinal samples of the lower respiratory tract collected every 3–6 days showed a high SARS-CoV-2 viral load (7.5 log_10_ RNA copies/100 cells), followed by a decrease to 1.3 log_10_ RNA copies/100 cells within 21 days (Figure 2, panel B). In contrast, lung *Legionella* DNA load increased and remained high (cycle threshold 21.9) until day 31. To identify potential bacterial co-infections, we performed a lung microbiota analysis on a D19 bronchoalveolar lavage using 16S MinION long-read sequencing technology (Oxford Nanopore Technologies, https://nanoporetech.com). Similar to another study of LD microbiomes (*13*), we found a predominance of *Legionella* (61%) and the presence of commensal lung bacteria (Appendix Figure) but no additional bacterial co-infection. On day 26, while high lung *Legionella* DNA load persisted, a third chest scan found pseudocavitation. Persistence of culture or PCR positivity in respiratory samples, or both, has been described in patients with *Legionella* lung abscesses, especially if immunocompromised (*14*).

## Conclusions

Our study found a substantial proportion of patients in LD notifications in France during March 2020, mostly elderly men with underlying conditions, also had COVID-19. They required ICU admission more frequently and had a higher case-fatality rate than patients without SARS-CoV-2 co-infection, but these rates were similar to that for all ICU-admitted LD patients (*7*,*10*). Overall, health effects from co-infections were more severe than from single infections, perhaps because of cumulative effects or because patients with co-infections may be more likely to have risk factors associated with poor outcomes. Another possibility is that SARS-CoV-2 infection may be more severe in this population. 

Longitudinal monitoring of a single co-infected patient found a first phase of predominant SARS-CoV-2 replication followed by a resurgence of *Legionella* and worsening of respiratory symptoms while SARS-CoV-2 decreased. Initial viral infection could establish pulmonary damage suitable for the bacteria to develop, similar to how bacterial superinfections develop in influenza-infected patients (*15*). Such co-infections may lead to poor prognoses as demonstrated here and elsewhere (*3*), underlining the importance of extensive screening for respiratory pathogens in patients with suspected or confirmed COVID-19. Because *Legionella* and other pulmonary microorganisms share clinical and radiological features with SARS-CoV-2 infection, they should be included in COVID-19 differential diagnoses (*3*). 

Appendix Additional information about study of co-infection with Legionnaires’ disease and coronavirus disease.
