# Supplementary material for: Co-infection with Legionella and SARS-CoV-2, France, March 2020
Source: Emerg Infect Dis. 2021 Nov;27(11):2864–8. doi: 10.3201/eid2711.202150 (PMC8544966; doi:10.3201/eid2711.202150)
Supplement: Appendix — Additional information about study of co-infection with Legionnaires’ disease and coronavirus disease. [file 20-2150-Techapp-s1.pdf]

Article DOI: https://doi.org/10.3201/eid2711.202150

Co-infection with *Legionella* and SARS-CoV-2, France, March 2020

Appendix

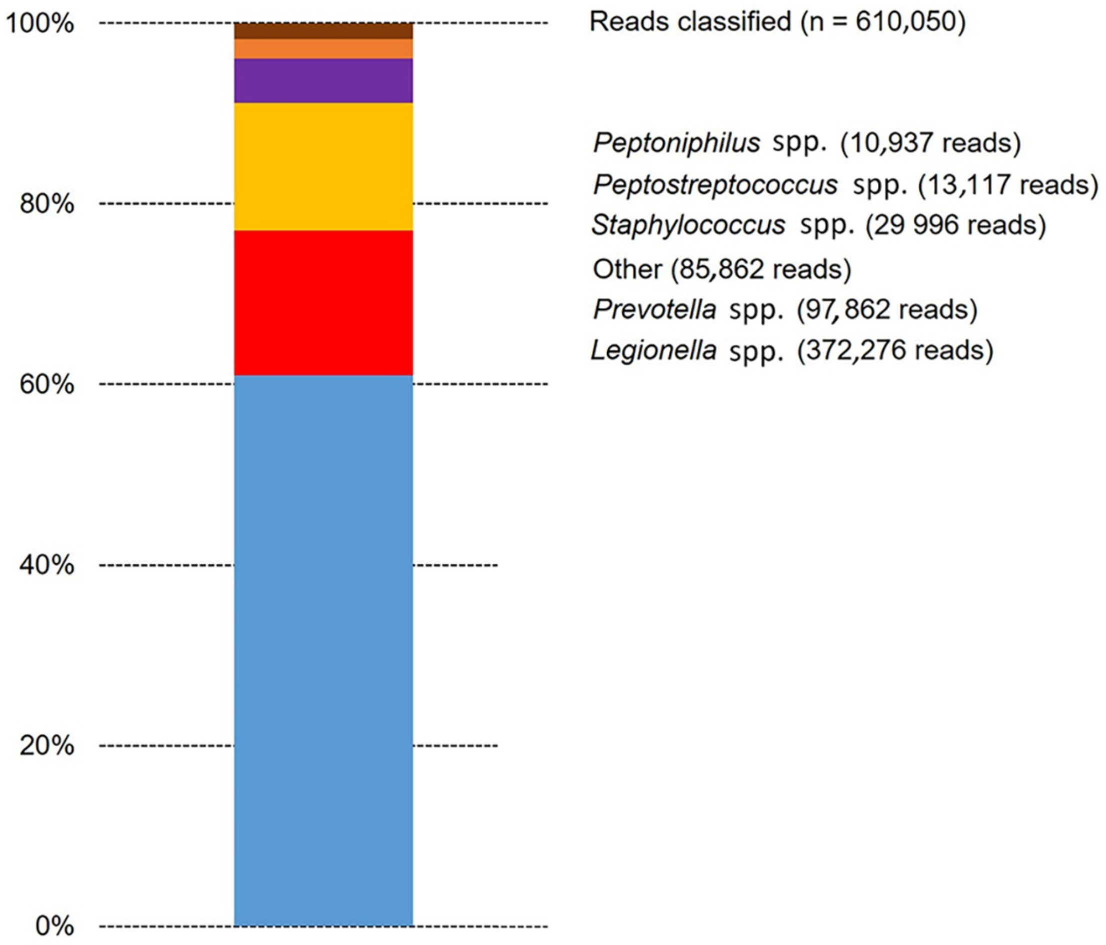

Appendix Figure. Bacterial composition of the D19 broncho-alveolar lavage sample. Results expressed as the percentage of bacterial genus among total classified reads. The taxonomy is based on the Epi2me workflow: taxonomic assignment for 16S amplicons (Oxford Nanopore, https://nanoporetech.com).
